# From Waste to Health: Upcycling Agrifood By-Products into Functional Antioxidant-Rich Ingredients and Products

**DOI:** 10.3390/antiox15091191

**Published:** 2026-09-18

**Authors:** Ítala M. G. Marx, Feliciano Priego-Capote

**Affiliations:** 1 Department of Analytical Chemistry, Campus of Rabanales, University of Córdoba, 14014 Cordoba, Spain; q72prcaf@uco.es; 2 Chemical Institute for Energy and Environment (IQUEMA), Campus of Rabanales, University of Córdoba, 14014 Cordoba, Spain; 3 Maimónides Institute for Biomedical Research (IMIBIC), Reina Sofía University Hospital, University of Córdoba, 14004 Cordoba, Spain; 4 Consortium for Biomedical Research in Frailty and Healthy Ageing (CIBERFES), Carlos III Health Institute, 28029 Madrid, Spain

## 1. Introduction

Over the past decade, agrifood by-products have undergone a remarkable transformation in scientific perception. Once regarded primarily as unavoidable waste streams requiring environmentally responsible disposal, they are now increasingly recognised as strategic sources of bioactive compounds capable of supporting the development of functional ingredients, nutraceuticals, cosmetics and other health-promoting products. This transition reflects a broader shift concerning more sustainable and resource-efficient food systems [1,2].

Early research largely focused on demonstrating the presence of antioxidant compounds and assessing their radical-scavenging capacity. While these studies established the scientific basis for agrifood by-product valorisation, they provided limited insight into biological relevance and translational potential. Today, the field increasingly integrates advanced analytical chemistry, sustainable extraction technologies, mechanistic biological validation and product development, showing the transition towards evidence-based functional ingredients.

This Special Issue brings together seven complementary contributions encompassing literature reviews, sustainable extraction technologies, advanced phytochemical characterisation, biological validation and product development. These studies illustrate the multidisciplinary nature of agrifood upcycling research while revealing the scientific and translational challenges that continue to shape the field.

This Editorial provides a critical synthesis of the collective advances emerging from this Special Issue, “*From Waste to Health: Upcycling Agrifood By-Products into Functional Antioxidant-Rich Ingredients and Products*”. By examining the studies as an integrated body of work, it identifies the major scientific trends, discusses the principal translational challenges and outlines future priorities for the development of evidence-based functional ingredients from agrifood by-products.

Together, these contributions indicate that agrifood by-product research is entering a new stage of scientific maturity [1,2,3,4,5,6,7]. Success will increasingly depend not on the simple recovery of antioxidant compounds, but on the generation of robust scientific evidence capable of supporting safe, effective and translational functional ingredients that contribute meaningfully to both human health and the circular bioeconomy.

## 2. Mapping the Scientific Landscape

The seven contributions included in this Special Issue encompass a range of biomass sources, technological approaches and application sectors, illustrating the multidisciplinary nature of contemporary agrifood by-product research. Together, they cover complementary stages of the innovation pipeline, from knowledge synthesis and biomass characterisation to sustainable extraction technologies, mechanistic biological validation and product development, as detailed in Table 1.

The published studies investigate diverse underutilised biological resources, including fruit by-products such as avocado peel and seed [1] and apple pomace [7], woody and plant-derived residues including *Camellia oleifera* shell [3] and *Glycyrrhiza glabra* leaves [5], marine processing by-products represented by sea cucumber viscera [4], and industrial side streams, such as pharmaceutical gelatine capsule waste [6]. This diversity illustrates the expanding definition of agrifood by-products, which now extends well beyond conventional agricultural residues to encompass a wide spectrum of renewable biological resources with potential applications in food, nutraceutical, cosmetic and biomedical sectors.

These studies span the principal stages required for the development of functional ingredients, including literature synthesis, sustainable processing, phytochemical characterisation, biological validation and product translation.

## 3. Emerging Trends and Challenges

The contributions assembled in this Special Issue illustrate a field undergoing rapid scientific evolution. Although they investigate distinct by-product sources and application sectors, several common themes emerge that reflect the increasing maturity of agrifood by-product research. These studies demonstrate a progressive shift from descriptive side-stream valorisation towards multidisciplinary strategies that integrate sustainable processing, advanced analytical chemistry, mechanistic biological validation and product development. At the same time, they expose important scientific and translational challenges that must be addressed before agrifood-derived ingredients can achieve widespread industrial implementation and regulatory acceptance.

### 3.1. Agrifood By-Products as Multifunctional Bioresources

One of the clearest trends emerging from this Special Issue is the growing recognition that agrifood by-products should no longer be regarded merely as repositories of antioxidant molecules. They represent complex biological matrices containing multiple classes of bioactive constituents, including polyphenols, flavonoids, polysaccharides, dietary fibre, minerals and other phytochemicals, that may interact synergistically to generate biological activity.

Nascimento et al. [1] and Estarriaga-Navarro et al. [2] emphasise the remarkable chemical diversity and multifunctionality of plant-derived by-products, highlighting opportunities across food, nutraceutical, cosmetic and biomedical applications. Complementing these reviews, Gao et al. [4] demonstrate that marine processing residues also represent multifunctional biological resources, while Chen et al. [3] and Serio et al. [5] illustrate how the selective recovery of specific phytochemical fractions can enhance biological performance. These studies indicate that the field is progressively embracing a systems-oriented perspective in which the overall composition of the biological matrix becomes a major determinant of functionality.

### 3.2. Green Extraction and Process Innovation

A second major trend concerns the evolution of extraction technologies. Maximising extraction yield is no longer the sole objective; increasing emphasis is now placed on extraction selectivity, preservation of bioactivity, environmental sustainability and industrial scalability.

This transition is evident in the studies by Chen et al. [3], who combined ultrasound-assisted extraction with resin purification to obtain highly purified multifunctional polyphenols, and Serio et al. [5], who employed supercritical CO_2_ extraction to selectively recover flavanone-enriched fractions with neuroprotective potential. Together with the technological advances discussed by Nascimento et al. [1], these studies demonstrate that extraction is evolving from a recovery process into a precision technology aimed at producing reproducible, standardised functional ingredients suitable for future industrial applications.

### 3.3. From Antioxidant Screening to Mechanism-Based Validation

One of the most important scientific transitions highlighted by this Special Issue is the movement beyond descriptive antioxidant evaluation towards mechanistic biological validation.

Several contributions integrate advanced chemical characterisation with cellular, animal and molecular models to investigate biological mechanisms. Chen et al. [3] demonstrated anti-inflammatory activity in macrophages, Serio et al. [5] explored neuroprotective properties associated with flavanone-enriched extracts, while Gao et al. [4] combined in vivo experimentation with gut microbiota profiling and activation of the Kelch-like ECH-associated protein 1 (Keap1)–nuclear factor erythroid 2-related factor 2 (Nrf2)/haem oxygenase-1 (HO-1) antioxidant signalling pathway. In parallel, Sanprasert et al. [6] and Szymczak et al. [7] incorporated metabolomic, functional and in vivo assessments into product development, illustrating the increasing integration of biological validation throughout the innovation pipeline. These studies substantially strengthen the biological relevance of agrifood-derived compounds and reflect the increasing demand for evidence capable of supporting future health claims and translational applications.

### 3.4. Remaining Barriers to Translation

Despite these advances, the contributions also reveal several common challenges that continue to limit scientific and industrial translation.

Methodological heterogeneity remains considerable, with substantial variability in extraction procedures, analytical platforms and biological assays. As discussed by Nascimento et al. [1] and Estarriaga-Navarro et al. [2], the absence of harmonised methodologies continues to hinder reproducibility and cross-study comparisons. Moreover, although studies [3,4,5,6,7] incorporate increasingly sophisticated biological models, none investigate the gastrointestinal fate of recovered compounds, including digestion, bioaccessibility, metabolism and bioavailability.

Similarly, while product research has advanced considerably through the development of functional foods and cosmetic formulations, clinical evidence remains scarce and regulatory aspects, including standardisation, toxicological assessment and quality control, are only marginally addressed. Finally, the diversity of phytochemical, biological and technological datasets generated across this Special Issue highlights the growing opportunity for artificial intelligence, machine learning and multi-omics approaches to optimise extraction processes, predict biological functionality and accelerate ingredient development.

Taken together, these observations indicate that the next phase of agrifood by-product research will depend not only on identifying new bioactive resources but also on generating robust, reproducible and physiologically relevant evidence capable of supporting commercial translation.

## 4. Conclusions and Future Perspectives

These contributions demonstrate that agrifood by-product research has reached an important stage of scientific maturity. Jointly, they illustrate the transition from conventional waste valorisation towards the development of evidence-based functional ingredients supported by sustainable extraction technologies, advanced phytochemical characterisation, mechanistic biological validation and product innovation.

Importantly, these studies also indicate that future progress will depend less on the discovery of new antioxidant-rich biomass and more on strengthening the translational pathway linking chemical composition to measurable health outcomes. Establishing harmonised analytical workflows, integrating physiologically relevant digestion and bioavailability models, generating robust clinical evidence and incorporating regulatory considerations from the earliest stages of product development will be essential to accelerate industrial implementation.

Future advances will also require closer integration of analytical chemistry, food science, nutrition, microbiology, engineering and computational sciences. Emerging tools such as artificial intelligence, machine learning and multi-omics offer unprecedented opportunities to connect chemical fingerprints with biological responses, optimise extraction strategies and support the rational design of next-generation functional ingredients.

Finally, the studies published in this Special Issue demonstrate that agrifood by-products are no longer viewed simply as waste streams requiring valorisation. Instead, they are increasingly recognised as strategic biological resources capable of supporting innovation across the food, nutraceutical, cosmetic and biomedical sectors. By highlighting both current achievements and remaining challenges, this Editorial provides a roadmap for future research and reinforces the central role of agrifood upcycling in advancing sustainable, health-oriented and circular food systems.

## Figures and Tables

**Table 1:** Overview of the studies included in this Special Issue and their main valorisation strategies, biological validation and applications.

Study	By-Product	Main Objective	Valorisation Strategy	Principal Bioactive Compounds	Level of Biological Validation	Main Application	Main Contribution
[1]	Avocado peel and seed	Summarise chemical composition, extraction technologies and functional potential	Critical review	Phenolic acids, flavonoids, tannins, starch and dietary fibre	Literature synthesis	Functional foods, nutraceuticals, packaging	Demonstrated that avocado by-products represent multifunctional ingredients but emphasised remaining challenges related to bioavailability, safety and industrial scalability.
[2]	Multiple plant by-products	Review current biomedical applications of plant residues	Critical review	Polyphenols, flavonoids, terpenoids, fibres and other phytochemicals	Literature synthesis	Biomedicine, dermocosmetics and nutraceuticals	Established the current state-of-the-art, highlighted translational challenges and identified research priorities for biomedical valorisation.
[3]	*Camellia oleifera* shell	Develop an efficient cascade process for polyphenol recovery	Ultrasound-assisted extraction + macroporous resin purification	Purified polyphenols (flavonoids and organic acids)	Chemical assays + cell-based anti-inflammatory model	Functional ingredients, cosmetics, food preservation	Demonstrated that process optimisation markedly improves extract purity and multifunctional bioactivity.
[4]	Sea cucumber viscera	Evaluate antioxidant potential under oxidative stress	Whole biomass valorisation	Polysaccharides, minerals and antioxidant compounds	In vitro assays + mouse model + gut microbiota + molecular pathway analysis	Functional food	Advanced beyond antioxidant screening by demonstrating modulation of the Keap1–Nrf2/HO-1 pathway and gut microbiota.
[6]	Pharmaceutical gelatine capsule waste	Develop a prebiotic functional jelly	Food reformulation using pharmaceutical gelatine capsule waste	Chamomile phytochemicals, inulin and polydextrose	Metabolomics + physicochemical characterisation + probiotic evaluation	Functional food	Demonstrated that pharmaceutical waste can be successfully transformed into consumer-ready functional foods with prebiotic properties.
[7]	Apple pomace + Roman chamomile hydrolate	Develop sustainable cosmetic ingredients	Cosmetic formulation using agricultural by-products	Polyphenols, phenolic acids and volatile compounds	Chemical characterisation + formulation + human skin evaluation	Dermocosmetics	Successfully translated agro-industrial residues into a cosmetic formulation with measurable skin performance.
[5]	*Glycyrrhiza glabra* leaves	Recover flavanone-enriched fractions using green technology	Supercritical CO_2_ extraction	Pinocembrin, licoflavanone, glabranin and related flavanones	Chemical characterisation + in vitro neuroprotective evaluation	Neuroprotective ingredients	Demonstrated selective recovery of high-value flavanones through solvent-free extraction while linking chemical composition to biological activity.

## Data Availability

No data was used for the research described in the article.
